# Recommendations for Researchers on Synchronous, Online, Nominal Group Sessions in Times of COVID-19: Fishbone Analysis

**DOI:** 10.2196/34539

**Published:** 2022-03-25

**Authors:** Lotte Timmermans, Ine Huybrechts, Peter Decat, Veerle Foulon, Ann Van Hecke, Mieke Vermandere, Birgitte Schoenmakers

**Affiliations:** 1 Academic Centre of General Practice KU Leuven Leuven Belgium; 2 Department of Family Medicine and Population Health University of Antwerp Antwerp Belgium; 3 General Practice and Primary Health Care Ghent University Ghent Belgium; 4 Clinical Pharmacology and Pharmacotherapy KU Leuven Leuven Belgium; 5 University Centre for Nursing and Midwifery Ghent University Ghent Belgium

**Keywords:** COVID-19, fishbone diagram, nominal group technique, video conferencing, primary health care, qualitative research

## Abstract

**Background:**

In times of COVID-19, we are challenged to experiment with alternative platforms or software to connect people. In particular, the struggle that arose in health research was how to interact with patients and care professionals. The latter is additionally faced with an extreme workload to fight the pandemic crisis. Creative strategies have been developed to continue research among patients and care professionals to improve quality of care. This paper addresses the issue of synchronous, online, nominal group sessions, a common consensus method used for group brainstorming.

**Objective:**

The purpose of this study was to share our experiences with performing online, nominal group sessions using the video conference software Microsoft Teams. In addition, we aimed to create a practical guide with recommendations for researchers.

**Methods:**

We critically analyzed the procedures for the online nominal group technique, according to the Fishbone methodology.

**Results:**

Performing synchronous, online, nominal group sessions is challenging but offers opportunities. Although interaction with and among the attendees complicates the process, the major advantage of online sessions is their accessibility and comfort because of reduced barriers to participation (eg, lower time investment). The role of the moderators is of major importance, and good preparation beforehand is required. Recommendations for future online, nominal research were formulated.

**Conclusions:**

Online, nominal group sessions seem to be a promising alternative for the real-life commonly used technique. Especially during the COVID-19 pandemic, the benefits must be highlighted. More expertise is needed to further refine the practical guide for using digital software in research and to achieve optimal performance.

## Introduction

The COVID-19 outbreak has posed multiple challenges. Not only has the pandemic caused major loss of human life globally, the impact on mental and psychosocial health should not be underestimated [1-4]. Apart from the major impact on human health, it also has an impact on health research. More specifically, the limited accessibility of research sources and participants obliges researchers to seek for alternatives to pursue research projects. Due to strict regulations to control transmission of the virus, researchers have to rely on creative strategies to involve the research population [5]. In-depth interviews and focus groups are conducted by using video conferencing software and platforms, but more complex group-based participatory methods have not yet been fully explored online [6-8]. The nominal group technique is one of these unexplored methodologies in research. This technique is commonly used to solve problems, determine priorities, or generate ideas [9]. The main challenge is cooperation in a strict and formal procedure [10]. Guidance is provided to a limited extent for face-to-face sessions, but the online setting remains unclear [9-11]. There are 2 different strategies for organizing online sessions: asynchronous sessions using an online platform to communicate in a nonsimultaneous way or synchronous sessions using video conference software in which participants join simultaneously. Since the nominal group methodology has not yet been extended fully online, very few researchers have utilized it [12,13]. Guidelines for recruiting participants, giving informed consent, and doing qualitative research with individuals are all based on the assumption of in-person interactions [14]. Therefore, there is a need to investigate in-depth the methodological issues of an online setup. In this paper, we describe the challenges and opportunities of the online form based on our experiences with 3 synchronous, virtual, nominal group sessions. In addition, participants’ experiences were explored. Finally, a practical guide with recommendations for researchers has been created.

The online sessions were part of a larger study by the Primary Care Academy (PCA) that aims to implement self-management support into primary care practice. Self-management is “the individual’s ability to manage the symptoms, treatment, physical and psychosocial consequences and lifestyle changes inherent in living with a chronic condition” [15]. Self-management of a chronic disease is a process in which health care professionals play a crucial supportive role [16-18]. The COVID-19 pandemic has highlighted the importance of self-management since the lockdown of many services resulted in more responsibility regarding handling of chronic diseases [19]. Online nominal group sessions were organized to brainstorm how health care professionals can support patients in this self-management process.

## Methods

We critically analyzed different aspects of synchronous, online, nominal group sessions, according to the Fishbone methodology. The nominal group sessions aimed to formulate specific actions to support patient’s self-management and were part of research to reinforce Flemish primary care.

### Data Collection

Data were collected during 3 online, nominal group sessions with health care professionals and experts by experience (ie, patients with knowledge based on personal experiences). Two qualified moderators conducted all the sessions, by use of the video conference software Microsoft Teams. With the participants’ consent, all 3 sessions were audio- and video-recorded and transcribed verbatim afterwards for qualitative analysis. At the end of each session, participants were offered the opportunity to share their experiences verbally, by use of the chat box, or by email afterwards.

### Online, Nominal Group Sessions

Similar to the classical nominal group technique, the online sessions were structured in 4 key stages: silent generation, round robin, clarification, and ranking [9]. A PowerPoint presentation was used to support this structure and the accompanying storyline. In advance, this slideshow was uploaded in Microsoft Teams instead of sharing the presentation, with the advantage of seeing the participants on the call. The protocol was developed specifically for online use and based upon comprehensive debate within the research group.

### Sampling and Recruitment

Maximum variation purposive sampling was used to recruit the nominal group participants by email or through health care organizations and practices. A registration link was embedded in a flyer. In addition, a call for participants was announced on the internet (website of the PCA). Participants included health care professionals and experts by experience from around Flanders. The aim was to have a minimum of 5 and a maximum of 10 participants per session [20].

### Procedure

Each session started with a short introduction, including explaining ground rules (which had also been communicated in advance by email) and a roundtable introduction by the attendees. Afterwards, information was provided on the topic of the session (self-management support) and the brainstorming procedure. These initial stages took 20 minutes and were followed by the active nominal group brainstorming. It started with the presentation of a specific question, related to self-management support, that gave rise to the formulation of ideas during the silent generation phase. This idea generation question (ie, “What concrete action/interventions can be designed to help patients fit chronic disease(s) into their lives in the most optimal way?”) was the impetus for brainstorming. Participants were asked in advance to have their pen and paper ready while brainstorming. To keep everyone’s attention, no more than 15 minutes were provided for generating ideas on self-management support. Additionally, the round robin stage in which ideas were exchanged filled in the next 20 minutes. Both moderators took notes during this stage. The formulated ideas were then rephrased by the moderators to make sure everyone gained a full understanding. Then, during a 15-minute clarification phase, there was time to clarify the ideas upon request of the participants. Finally, the nominal group sessions ended with a ranking stage of 5 minutes to 10 minutes, depending on the number of ideas formulated. More specifically, a ranking system created with the online service “Poll Everywhere” (San Francisco, CA) was included in the slideshow to prioritize the ideas generated according to the participants’ preferences. A short break of 10 minutes after the generation of ideas gave the moderators the opportunity to process them in the ranking system. The second and third sessions had an additional phase just before the polling in which the ideas previously formulated were quickly reviewed and included in the ranking system. Each session was planned to last no longer than 1.5 hours.

### Analysis of the Content Related to Self-Management Support

Processing the output of the brainstorming sessions (ie, formulated ideas on self-management support) was performed analogous to the regular methods of the nominal group technique. In summary, the generated ideas were (after performing 3 brainstorming sessions) organized in themes by qualitative coding of the transcripts and processed in a survey. Participants were asked to identify their favorite idea and to rank the ideas based on their preferences by defined theme. Afterwards, ideas were grouped by the main researcher (LT) following a self-management support model into 5 categories according to the type of primary care action (ie, supporting, involving, listening to, coordinating, or questioning patients). Ideas that were not chosen as favorites were excluded. Subsequently, the table was reviewed by the research team during multiple rounds to reach a consensus on the categorization. Finally, ideas were further refined according to the survey results and processed for use in a primary care practice intervention related to self-management support. The results of the analysis related to the formulated ideas of the online sessions are beyond the scope of this research paper and will be reported elsewhere.

### Ethical Approval

Ethical approval for the brainstorming sessions was obtained from The Ethics Committee Research UZ/KU Leuven (S63890). An informed consent document was sent to the participants in advance by email. It explicitly stated that conversations were video- and audio-recorded during the online sessions. In addition, participants were informed that the content remained internal for scientific research purposes.

The return of a signed copy was required to participate in the online sessions. After confirmation of participation and signing the informed consent form, attendees obtained an invitation link to a Microsoft Teams live event. Access to the recorded sessions (audio, video, and shared slideshow) was limited to the research group. The entire study was conducted in accordance with the Helsinki Declaration.

### Outcome Measures

We defined 6 different process outcomes to characterize the online nominal group sessions: guidance, engagement, interaction, timing, technology, and participants’ experiences. These categories were initially described by the main researcher based on literature analysis and further refined by the research team.

### Analysis of the Online, Nominal Group Procedure

Audio and video recordings, together with written data (ie, transcripts, Teams chat, and email data) from the nominal group procedure, were analyzed for the purpose of this study. A deductive “top-down” approach was applied to collect meaningful data from these sources, by exploring the predefined outcomes. More specifically, a Fishbone diagram was used to explore all possible challenges researchers and participants faced when performing or attending the online, nominal group sessions, as this methodology helps team members visualize the potential problem sources. The analysis starts by defining a central problem, which is then deeply examined by formulating several causes, organized in different categories [21]. A literature search revealed that an online, nominal group technique has rarely been performed (and reported); only very few research articles explored this online methodology. This resulted in the definition of our central problem: low uptake of synchronous, online, nominal group sessions in research practice. The final Fishbone diagram incorporated in the study was reviewed by the entire research team to manage bias and ensure the validy of our findings.

### Trustworthiness

To ensure rigor, we applied different strategies to increase trustworthiness of the data. First, maximum variation sampling was applied to recruit the participants for the nominal group sessions. Participants of different ages, sexes, regions, educational levels, and employment were chosen. Second, the 3 nominal group sessions were fully recorded and transcribed verbatim. In addition, field notes were taken. Third, dependability of the findings was established by providing a detailed description of the online, nominal group procedure. Finally, the analysis was checked by qualitative experts in the field.

### Research Team

LT and IH performed the online nominal group sessions and collected the data. LT analyzed the data and designed the Fishbone diagram, in close collaboration with supervisor BS. The entire research team (IH, PD, VF, AVH, MV, and BS) reviewed the final diagram that was incorporated in this manuscript. This group consists of experienced members in qualitative health research. Before project initiation, the moderators received additional intensive training on the principles and methods in qualitative research to assure a certain level of standardization. Both moderators had previous expertise in quantitative and qualitative data collection in a group setting.

## Results

After performing 3 synchronous, online, nominal group sessions in which individuals brainstormed on specific actions to support patients’ self-management, a Fishbone analysis was undertaken. A total of 24 persons participated in the online sessions and therefore contributed to the data collection.

### Challenges for Online, Nominal Group Sessions

**Figure 1:**
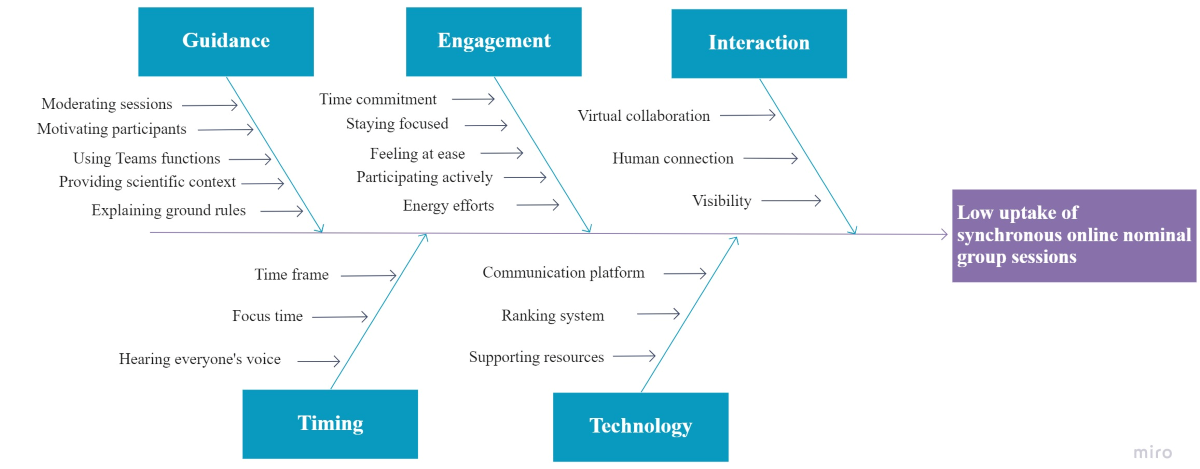
Fishbone diagram representing identified causes challenging the uptake of synchronous, online, nominal group sessions. Template retrieved from an online collaborative whiteboard [22].

Various causes were identified that positively or negatively challenged the online performance. These causes were clustered under the predefined outcome measures: guidance, engagement, interaction, timing, technology, and participants’ experiences. Each outcome measure was thoroughly examined by audio and video analysis, supplemented with input from the chat and email, to identify the potential causes of the low uptake of online, nominal group sessions in research practice. Figure 1 represents the final Fishbone diagram.

#### Guidance

Moderating the online sessions challenged the research team in various ways. The moderators’ role was of major importance since they had to guide the discussion in an online setting. The main barrier was managing participants during the brainstorming while not being able to interact directly, neither verbally nor nonverbally. Participants were encouraged to provide input by elevating a virtual hand using the hand icon of Microsoft Teams. A moderator then appointed the person to speak at an appropriate time during the session. The importance of guiding participants was especially expressed during the round robin and clarification stages. To ensure everyone could provide their input, participants were indicated one by one to express their ideas on the topic of self-management. Examples of formulated ideas included the following: introducing a buddy system among peers, organizing interactive sessions on chronic disease coping strategies, integrating communication aids and strategies in primary care practice. This structured approach while exchanging the ideas seemed to have positively contributed to an equal involvement of all participants, even those who had told us beforehand they were not very articulate. Questions to each other when sharing ideas could often not be answered immediately. In these cases, the use of a chat box could act as an intermediate communication medium. Although instructed, this medium was rarely used by the participants.

During the entire nominal group session, attention was paid to expressing the scientific context and methodology in simple terms—for example, the moderators did not use the word self-management itself when guiding conversations and asking the idea generation question. This resulted in an overall understanding of the background information, setup, and aim of the nominal group technique. Based on the participants’ feedback we received, we can conclude that the absence of real-life interaction emphasized the importance of strictly guided sessions. Also, clear instructions before and at the start were necessary given the simultaneous use of the chat box, hand icon, and PowerPoint presentation by the moderator, while interacting with the audience.

#### Engagement

During the sessions, engaging participants was challenging because of the online setup. Some actions by the moderators were observed as a remarkable help, since they triggered the audience to contribute to the conversation. First, the sessions began with a roundtable introduction, encouraging participants to get to know each other in an accessible way. This introduction revealed a variety of backgrounds: project officers of care organizations, general practitioners, nurses, patients with chronic conditions and informal caregivers, representatives from patient associations, lecturers, and researchers in primary care. Second, the use of informative PowerPoint slides during the idea generation phase seemed a welcome addition, since all participants indicated they had a full understanding of the brainstorming procedure after reading a concise information sheet on the general topic and the idea generation question. Third, to give participants time to think and feel at ease, they were asked to switch off the camera during the silent idea generation phase. Fourth, regarding the engagement while exchanging ideas, the use of a round-robin questioning format allowed each participant to provide input. Attendees were motivated to actively participate by emphasizing that all input was valuable. However, we noticed the online setup demanded extra effort from the participants. This was mainly observed at the end of the sessions, when there was reduced active contribution to the brainstorming. This could be attributed to the long period of attention on the computer screen.

#### Interaction

The online design challenged the interactive process of brainstorming since participants could not rely on human interaction and the use of sticky notes, both key elements in real-life nominal group sessions. Nevertheless, some elements did contribute to a relatively vibrant process. Slides with strong visuals, including images to supplement content, engaged participants online and resulted in virtual collaboration. Participants also used pen and paper to write down their thoughts. In addition, the hand icon was used to interact with the research team. Unfortunately, active interaction or conversation among participants was not enhanced by the online setup. The only stage in which participants were able to communicate with each other in a coordinated way was the clarification stage.

A remarkable aspect of the online setup was how the camera use influenced the level of interaction. A sense of interconnectedness was created when participants were asked to switch on their webcams, determined by the observation that attention increased and the conversation became more lively. Noteworthy is that when sharing the PowerPoint presentation, this connected feeling diminished as the screen was taken up by the shared slides instead of the video streams. To overcome this barrier, screensharing was interrupted while actively brainstorming during the round robin and clarification stages.

#### Timing

Overall, the timing of the virtual sessions turned out well, although moderators were challenged to stick to the schedule in the foreseen period. To maintain the timetable, the entire nominal group session was precisely structured, and every part was delineated according to a strict time frame. The timing was tuned to the PowerPoint slides. For the moderators, staying within the time frame was rather challenging since they had to find a balance between providing enough time versus sticking to the strict time frame. To make sure sessions were not too strenuous, a break of 10 minutes was incorporated in between and appeared to be sufficient. However, the time frame was very tight, resulting in a rushed feeling among the moderators and reduced room for unforeseen circumstances (eg, delayed start). We also noticed that there was not enough time to prioritize the ideas with the external ranking system. To resolve this, we provided the opportunity to rank immediately afterwards up to a few hours after each session. However, this function was rarely used.

#### Technology

The online nominal group design challenged the participants since they had to have access to reliable technology to successfully engage within the sessions. In addition, necessary skills for using the various instruments (ie, Microsoft Teams, Poll Everywhere) were expected. Likewise, the moderators had to be able to manage the interaction with the audience, processing of ideas (ie, submitting in a ranking system), and navigation through the slideshow. This emphasized the need for guidance from 2 moderators. Unfortunately, due to the large number of participants (between 7 and 10 in each session), not all the faces were visible at the video bar while presenting the slides. This was a disadvantage because visual interaction appeared to contribute to participants’ engagement.

### Participants’ Experiences

I would like to compliment you (ie, the main moderator/researcher) on the way you took the lead in the session. You gave enough guidance so that it was pleasant to follow at a smooth pace. That is not always evident online.

Participant

Based on voluntary feedback in the chat, by email, or orally, participants’ experiences regarding the online sessions were positive overall. Furthermore, participants explicitly indicated they wished to cooperate further on the research project. The only negative feedback was received regarding the large number of ideas that had to be prioritized using the ranking system (Poll Everywhere). Participants verbally indicated that they did not have enough time to rank the ideas in the foreseen time frame. This resulted in confusion for some participants and failure to reach a full consensus at the end of each session, since consensus can only be reached when everyone gives their input. It is possible that participants preferred to have more time in a relaxed atmosphere to rank ideas. This resulted in the development of an additional short survey in Qualtrics (Provo, UT) after completion of the 3 sessions, in which all participants had the opportunity to cast their vote on the entire group of ideas (total of 3 sessions).

## Discussion

### Practical Guide With Recommendations

**Figure 2:**
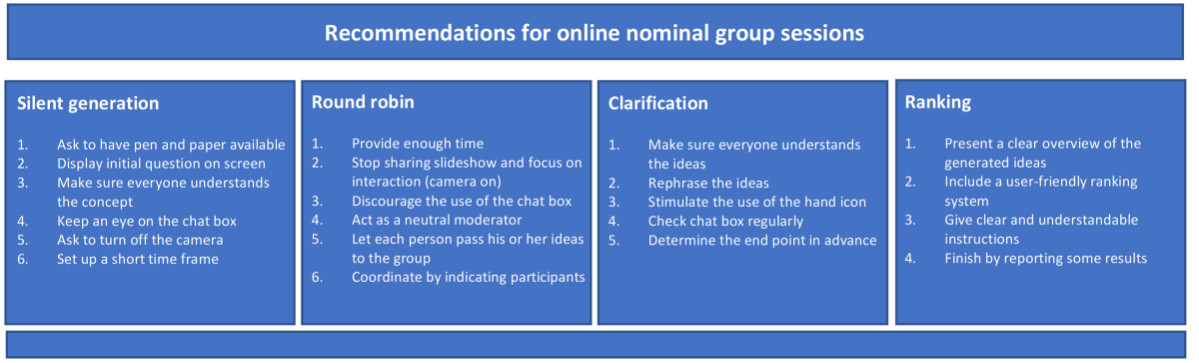
Recommendations for planning and implementing synchronous, online, nominal group sessions.

Guidance for performing online nominal group studies is missing. However, it has been stated that results of the same quality can be achieved with an online approach [23]. In addition, studies show that there is no specific need to perform real-life sessions [13]. We used a Fishbone diagram to identify all possible challenges that researchers face when performing online, nominal group sessions. Based on our findings and experiences with the online setting, we developed a practical guide with recommendations for researchers interested in this type of work. The recommendations were pooled into the main stages of the nominal group: silent generation, round robin, clarification, and ranking (Figure 2). In addition, some general recommendations were formulated.

#### Silent Generation

At the beginning of the session, the moderators should clearly explain the concept. During the brainstorming, pen and paper are indispensable. Taking notes helps the participants to explore ideas. Instead of classical notes, an online platform or whiteboard can help participants brainstorm but can be more complicated. Displaying the initial question on the screen during the generation process adds value. We recommend keeping this phase short, to avoid losing someone’s attention. Moderators should encourage participants to use the chat box when personal issues occur. Oral interventions are not recommended. This results in disruption of the individual reflection. An exception should be made for communications that are relevant to the whole group. In addition, switching off the cameras also contributes to thinking in serenity. However, disabling cameras can be discussed as a negative element, as participant’s behavior can no longer be interpreted. In addition, seeing each other visually can result in increased commitment to the idea generation.

#### Round Robin

To engage participants during the round robin, we recommend stopping screen sharing. Switching on the video camera leads to a more interactive conversation. To further increase the level of interaction, try to minimize the use of the chat box while exchanging ideas. If still used during this stage, motivate the chat box user to ask the question out loud. We suggest providing enough time to exchange the ideas generated so that everyone can have their say and the moderators have time to take notes. Furthermore, it is essential that moderators act as neutral as possible to avoid judgment and criticism. They should monitor the participants’ engagement. Coordination is crucial for every person to pass their ideas to the group.

#### Clarification

Collaboration and interaction are highly affected in the online setup since there is less nonverbal communication. This challenges participants to speak more out loud during the clarification round. Unfortunately, not everyone feels comfortable doing so. The use of the chat box as an intermediate communication platform can counteract this but was rarely used. Moderators should make sure everyone understands the ideas formulated. It helps to reformulate ideas in different ways and to check that those present understand them. Make sure the use of the Microsoft Teams hand icon is encouraged to avoid a chaotic conversation. Analogous to the regular methods of the nominal group technique, we advise determining the end point of this research phase in advance to prevent clarification from becoming an ongoing process.

#### Ranking

Some participants are overwhelmed by a multitude of information. Providing a clear overview of the generated ideas at the beginning of the ranking process is helpful. This procedure should be user-friendly, and instructions should be incorporated into the accompanying slideshow. The time it took to rank the ideas generated seemed to be person-dependent. Therefore, it is valuable to familiarize oneself with the audience while preparing the online sessions. To finish the brainstorming in a pleasant way, let the participants take a sneak peek at the results. This can be done by reporting the most favorite idea so far (ie, ranked as number 1).

### General Recommendations

Organizing synchronous, online, nominal group sessions requires thorough preparation. Guidance by at least two moderators is necessary. Due to the online complexity, moderators can benefit from special training. Not only are skills required from the research team but participants also need to develop online competencies. Unfortunately, not everyone is able to deal with online platforms. In Belgium, more than 15% of the adult population has a low level of health literacy, and in addition, 10% of households do not have an internet connection [24,25]. This must be considered when setting up online research with lay participants. Communication with the participants is essential to operate efficiently. More specifically, moderators should provide clear instructions before, at the start of, and during sessions. Furthermore, it is important to make a detailed schedule and monitor time. To engage participants as much as possible, more emphasis should be put on enhancing human interaction. The format of the nominal group technique is well adapted to empower attendees [13,26]. However, it should be mentioned that due to a strict pattern and guidelines, the nominal group technique never allows a lot of interaction. Not being able to ask each other questions immediately, due to the strict design, might also challenge participants’ patience. Starting with a roundtable introduction, switching on the video cameras, and using interactive slides with visual components seem to make attendees feel comfortable. In addition, the possibility can be offered to contact the research team afterwards. Researchers should aim for a meeting moment that works best for the target population, and the importance of attending at the scheduled time needs to be emphasized.

Computer screens have been proven to negatively influence the activity of the human brain [27]. Therefore, the duration of the meeting should be limited. By contrast, online sessions are less subject to distractions, which means less time is lost and researchers can better stick to the schedule. Unfortunately, the strict timing can result in a rushed feeling. In our opinion, this cannot be compensated by allowing more time for the brainstorming, as we noticed focus decreased the longer the session lasted. A second break could be a possible solution.

The main advantage of performing online sessions seems to be the flexibility and comfort for both researchers and attendees. The online setup increased the accessibility for participants who otherwise might have experienced barriers to participation, such as feelings of discomfort in a group setting, transportation issues, or time [13]. Especially for health care professionals, who were in the middle of the battle during COVID-19 pandemic, it was a great opportunity to actively participate in research. The flexibility might explain the large turnout and few cancellations. This is in striking contrast to real-life sessions in which organizational issues seem the main limitation [10].

### Strengths and Limitations

Some limitations should be mentioned. First, performing only 3 online sessions limited the generalizability of the results. However, after performing 3 sessions, we reached a point of saturation regarding both the findings on the topic of self-management support and on the methodological challenges and opportunities. Moreover, we did not compare our findings with real-life nominal group sessions. This paper only elaborates on the synchronous, online technique. Based on the performance of 3 sessions, we were able to gain sufficient input to analyze challenges. Knowledge of the real-life setup was gained through literature analysis. Furthermore, participants’ experiences were not deeply surveyed or explored. However, we achieved a notion of participants’ satisfaction by voluntary feedback in the chat, by mail, or orally. In addition, the research team reflected critically about the objectivity of the observations during multiple review rounds. Finally, the use of sophisticated online brainstorming tools was not included in our brainstorming procedure. These platforms can increase engagement during brainstorming. However, we deliberately chose simple operating systems (ie, Microsoft Teams, Poll Everywhere), because not everyone is able to work with such tools. By choosing low-threshold systems, a wide target group could participate in the online, nominal group sessions.

### Practical Implications

Online, nominal group sessions seem to be a promising alternative to the commonly used real-life technique. This paper provides researchers with recommendations for conducting online sessions, considering the various challenges. In our experience, the online format is highly recommended when looking for research procedures that are very accessible and consume little time. Especially during the COVID-19 pandemic, the benefits must be highlighted.

### Future Research

Future research should focus on refining the online, nominal group technique. More expertise is needed to optimize the practical guide and to achieve optimal performance. Comparative studies between the real-life and virtual setups are required to make statements about the efficiency. Researchers should consider participants’ experiences in the design of future online sessions.

### Conclusion

Performing synchronous, online, nominal group sessions is challenging but offers opportunities. One should be aware of the differences between real-life and online sessions. Good preparation is needed to overcome the barriers of the online technique. Our practical guide for researchers offers recommendations to facilitate the process. The role of the moderators is of major importance during brainstorming. Further research should refine the online procedure and make it more accessible for both researchers and the research population.

## Abbreviations

PCA: Primary Care Academy
